# Regulation of Cytosolic pH: The Contributions of Plant Plasma Membrane H^+^-ATPases and Multiple Transporters

**DOI:** 10.3390/ijms222312998

**Published:** 2021-11-30

**Authors:** Jin-Yan Zhou, Dong-Li Hao, Guang-Zhe Yang

**Affiliations:** 1 Jiangsu Vocational College of Agriculture and Forest, Jurong 212400, China; 23240962@163.com; 2 The National Forestry and Grassland Administration Engineering Research Center for Germplasm Innovation and Utilization of Warm-Season Turfgrasses, Institute of Botany, Jiangsu Province and Chinese Academy of Sciences, Nanjing 210014, China; 3 State Key Laboratory for Conservation and Utilization of Subtropical Agro-Bioresources, College of Life Science and Technology, Guangxi University, Nanning 530004, China; ygz201@163.com

**Keywords:** H^+^ transport proteins, cytosolic pH homeostasis, H^+^ transfer pathway, pH regulation

## Abstract

Cytosolic pH homeostasis is a precondition for the normal growth and stress responses in plants, and H^+^ flux across the plasma membrane is essential for cytoplasmic pH control. Hence, this review focuses on seven types of proteins that possess direct H^+^ transport activity, namely, H^+^-ATPase, NHX, CHX, AMT, NRT, PHT, and KT/HAK/KUP, to summarize their plasma-membrane-located family members, the effect of corresponding gene knockout and/or overexpression on cytosolic pH, the H^+^ transport pathway, and their functional regulation by the extracellular/cytosolic pH. In general, H^+^-ATPases mediate H^+^ extrusion, whereas most members of other six proteins mediate H^+^ influx, thus contributing to cytosolic pH homeostasis by directly modulating H^+^ flux across the plasma membrane. The fact that some AMTs/NRTs mediate H^+^-coupled substrate influx, whereas other intra-family members facilitate H^+^-uncoupled substrate transport, demonstrates that not all plasma membrane transporters possess H^+^-coupled substrate transport mechanisms, and using the transport mechanism of a protein to represent the case of the entire family is not suitable. The transport activity of these proteins is regulated by extracellular and/or cytosolic pH, with different structural bases for H^+^ transfer among these seven types of proteins. Notably, intra-family members possess distinct pH regulatory characterization and underlying residues for H^+^ transfer. This review is anticipated to facilitate the understanding of the molecular basis for cytosolic pH homeostasis. Despite this progress, the strategy of their cooperation for cytosolic pH homeostasis needs further investigation.

## 1. Introduction

As a fundamental activity in all living cells [1], cytosolic pH homeostasis is essential for the normal growth and stress responses of plants [2,3]. This is because basic cytosolic processes such as biochemical reactions, protein stability, ion channel/transporter activity, compartmental integrity, and membrane trafficking have strict pH requirements [1,4]. Simultaneously, most protein machineries (enzymes, motors, vesicle traffic, ribosomes, spliceosomes, assembly proteins, regulators, etc.) can only work within a narrow pH range [5]. Studies have shown that plant cytosolic pH is stable at a small range of 7.1–7.5 [5,6,7,8].

Cytosolic pH homeostasis is mainly controlled by the following three factors: first, chemical buffering components which comprise bicarbonate, phosphate, protein buffers (e.g., the imidazol group of histidine), etc. [9,10,11,12]; second, cytosolic H^+^ consumption and H^+^ generation by metabolism [5,8,13]; and third, the direct H^+^ flux across the plasma membrane and endomembrane [1,7,12,14,15,16].

In comparison with numerous reviews that concentrate on the organelle-located proteins which are responsible for H^+^ flux across the endomembrane [1,6,7], summaries regarding proteins that are directly involved in H^+^ efflux/influx across the plasma membrane are scarce, except regarding plasma membrane H^+^-ATPases [14,17]. However, the topic with which we are concerned is not included in the two above-mentioned studies. Thus, this review focuses on seven types of proteins that possess direct H^+^ transport activity, namely, H^+^-ATPase (H^+^-pumping ATPase), NHX (Na^+^/H^+^ exchanger), CHX (cation/H^+^ exchanger), AMT (ammonium transporter), NRT (nitrate transporter), PHT (phosphate transporter), and KT/HAK/KUP (K^+^ transporter/high-affinity K^+^ transporter/K^+^ uptake permease), to summarize their plasma-membrane-located family members, the effect of changes in their transcript levels on extracellular/cytosolic pH, the H^+^ transport mechanism, and their functional regulation by either extracellular or cytosolic pH. Finally, prospects are presented in this field with emphasis on the necessity to determine the cooperative strategy of these proteins for cytosolic pH homeostasis.

## 2. Roles of Plasma Membrane H^+^-ATPases and Multiple Transporters in Cytosolic pH Homeostasis

### 2.1. H^+^-ATPase Family

#### 2.1.1. Plasma-Membrane-Located Family Members, Function and the Effect of Their Expression Level Changes on the Cytosolic pH

Plant plasma membrane H^+^-ATPases (H^+^-pumping ATPase) have many family members. This notion is supported by the fact that 10 plasma membrane H^+^-ATPases have been found in the model plant Arabidopsis genome [14,18,19]: 10 in rice [20], 12 in tomato [21], 4 in maize [22], 8 in *Marchantia polymorpha* [23], 10 in cucumber [24], 7 in potato [25], 9 in tobacco [26], and 13 in sunflower [27] genomes.

Plant plasma membrane H^+^-ATPases actively pump H^+^ from the cytoplasm to the extracellular space using the energy generated by ATP hydrolysis; thus, they are essential for cytosolic pH homeostasis. The process of H^+^ efflux is not accompanied by other ions [17,28,29,30]. The effect of plasma membrane H^+^-ATPases’ activity and/or expression level changes on the cytosolic pH is mainly reflected by the measurement of indirect extracellular pH variation. Firstly, pharmacological test results show that the addition of a strong H^+^-ATPases activator, Fungal Toxin Fusicoccin, results in the acidification of tomato culture growth medium [31,32], whereas the inclusion of H^+^-ATPases activity inhibitors (such as Erythrosin B or diethyl stilbestrol) leads to the alkalization of growth media [31]. Secondly, the expression of either single NpPMA2 (*Nicotiana plumbaginifolia* plasma membrane H^+^-ATPase 2) or NpPMA4 (*Nicotiana plumbaginifolia* plasma membrane H^+^-ATPase 4) in the heterologous yeast system leads to acidification of the growth medium [33]. Thirdly, in planta measurements through knockout and/or overexpression materials. Overexpression of an active isoform of AHA3 (T948D-AHA3, a mutant with T to D alternation at position 948 of Arabidopsis H^+^-ATPase 3) enhances the tolerance of Arabidopsis to acid stress, a phenomenon which is consistent with its roles in the extrusion of toxic H^+^ from the cytoplasm [34]. Overexpression of rice OSA1 (*Oryza sativa* plasma membrane H^+^-ATPase 1) leads to a ~1 unit decrease in the growth medium pH [35]. As two main H^+^-ATPases in Arabidopsis roots [36,37], the single knockout of AHA2 (Arabidopsis H^+^-ATPase 2) quantitatively results in a ~1 unit increase in the growth medium pH [37], and the single knockout of AHA1 (Arabidopsis H^+^-ATPase 1) causes a 60% reduction in the H^+^ efflux capacity in planta [38]. The single knockout of AHA7 (Arabidopsis H^+^-ATPase 7) also significantly reduces the H^+^ efflux capacity in the root hair zone under low-phosphorus stress [39]. All these results indicate the contribution of plasma membrane H^+^-ATPases to cytosolic pH control, but the direct measurement of cytosolic pH changes upon their mutation/overexpression is still lacking. Until recently, the observation that the triple knockout of AHA6/8/9 (Arabidopsis H^+^-ATPase 6/8/9) results in a ~0.5 unit decrease in the cytosolic pH has preliminarily quantified its role in cytosolic pH homeostasis [40].

#### 2.1.2. Mechanism of H^+^ Transport

Results from AHA2 facilitate the understanding of the H^+^ extrusion pathway. It is proposed that a single, centrally located proton acceptor/donor (D684), an asparagine residue (N106), a positively charged arginine residue (R655), and a large central cavity form the H^+^ transporting unit [41]. The H^+^ transfer process can be divided into two steps: the H^+^ loading and release. Briefly, the side chain of a conserved D684 residue receives the proton, causing protonation of this residue. Then, the protonated form of D684 forms an occluded and hydrogen-bonded pair with the equally conserved N106 residue [29,42]. Subsequently, conformational movements trigger the opening of the proton exit cavity and the interruption of hydrogen bonding between N106 and D684, finally leading to proton release from D684 [41,42]. The conserved R655 is proposed to favor the release of the bound H^+^, possibly through polarizing the D684 side chain and modulating its pKa [41,42,43].

#### 2.1.3. Regulation by Extracellular/Cytosolic pH

Activation of AHA7 occurs only when the extracellular pH is ≥6.0. Sensing of the extracellular pH is controlled by the extracellular loop between transmembrane segments 7 and 8 [44].

The relationship between the plasma membrane H^+^-ATPase and the cytosolic pH shows a “bell” shape, with the maximal transport activity occurring at around pH 6.5 [33,40,45,46]. For instance, the optimum pH for the H^+^-ATPase activity of a plasma membrane fraction from Arabidopsis is 6.6 [47], whereas the optimum pH for that from rice is 6.0 [48]. The observation that low pH treatment enhances the transport activity of plasma membrane H^+^-ATPase in rice and soybean under hydroponic conditions is interpreted as the result of cytosolic acidification [49,50]. In a wide pH range, a one-unit decrease in the extracellular pH would lead to a 0.1 reduction in the cytosolic pH [51,52]. As mentioned above, the cytosolic pH is generally 7.4, and the optimum pH for H^+^-ATPase activity is around 6.5. Thus, the cytosolic acidification caused by the low-pH treatment may enhance the activity of H^+^-ATPase by shifting cytosolic pH towards its optimum pH [53].

### 2.2. NHX Family

#### 2.2.1. Plasma-Membrane-Located Family Members, Function and the Effect of Their Expression Level Changes on the Cytosolic pH

Amongst eight NHXs (Na^+^/H^+^ exchanger) in Arabidopsis [54,55], only two genes (AtNHX7 and AtNHX8) are located in the plasma membrane [56,57,58,59]. Homologues of the AtNHX7 widely exist in plants such as wheat, maize, and tomato [60,61,62]; however, no protein homologous to AtNHX8 has been found in the sequenced genomes of cereals [63].

The Arabidopsis AtNHX7/SOS1 (*Arabidopsis thaliana* Na^+^/H^+^ exchanger 7/Salt Overly Sensitive 1) functions as a plasma membrane Na^+^/H^+^ antiporter [56,64]. This protein mediates the efflux of Na^+^ out of the cytoplasm to the extracellular space, and exchanges equivalent H^+^ influx into the cytoplasm [7,65,66,67]. Thus, NHX genes are involved in cytosolic pH homeostasis [68,69]. Studies have demonstrated that the knockout of SOS1 in Arabidopsis and rice results in ~80% or ~40% reductions in the Na^+^/H^+^ exchange activity in plasma membrane vesicles, respectively, relative to activity in wild-type plants [70,71]. Upon NaCl treatment, the knockout of SOS1 reduces the capacity of H^+^ influx into the cytoplasm and results in cytosolic alkalization [72,73].

AtNHX8 is proposed to function as a Li^+^/H^+^ exchanger [54]. Observations indicate that the knockout of AtNHX8 renders the plants more sensitive to Li^+^, whereas overexpression of this gene enables the plant to be more tolerant to Li^+^, confirming the contribution of this gene to Li^+^ extrusion [54]. However, direct experimental evidence involving H^+^ influx by AtNHX8 is still lacking.

#### 2.2.2. Mechanism of H^+^ Transport

Interpretation of a lower resolution (25 Å) crystal structure of SOS1 demonstrates that it is a homodimer, which contains a membrane domain and an elongated, large, and structured cytosolic domain [74]. To illustrate detailed mechanisms for H^+^ transport, higher-resolution structural techniques are necessary [74,75].

#### 2.2.3. Regulation by Extracellular/Cytosolic pH

Knockout of the SOS1 transporter of *Physcomitrella patens* (PpSOS1) results in the enhanced influx capacity of Na^+^ at pH 4.5, but not for that at pH 9.0, suggesting that the transport activity of PpSOS1 is stimulated by low extracellular pH [76]. This acid-facilitated transport activity is in consistent with its Na^+^/H^+^ antiport function.

### 2.3. CHX Family

Amongst 28 members of CHXs (cation/H^+^ exchanger) in the Arabidopsis genome [77,78], AtCHX13 [79], AtCHX14 [80], AtCHX16 [81], AtCHX17 [81], AtCHX18 [81], AtCHX19 [81,82], and AtCHX21 [83] have been found to be localized in the plasma membrane. Three-dimensional homology modeling and point mutation results indicate that AtCHX17 has a core structure similar to Na^+^/H^+^ antiporter [84]. It is thus proposed that AtCHX17 contributes to cytosolic pH homeostasis by mediating H^+^ influx across the plasma membrane. Expressions of AtCHX16–AtCHX19 in a yeast mutant defective in Na^+^ extrusion and K^+^(Na^+^)/H^+^ antiport rescue the alkaline pH-sensitive growth phenotype, also supporting their potential roles in cytosolic pH homeostasis [85]. However, to date, the H^+^-coupled transport mechanisms of these plasma membrane CHXs have not been evidenced by direct experiments [7]. Notably, AtCHX13 is proposed to be a K^+^-uptake transporter [79], but AtCHX14 is expected to be a K^+^-efflux transporter [80]. This phenomenon suggests that the CHX intra-family may possess a distinct H^+^-related transport mechanism, which should be assessed with caution.

### 2.4. AMT Family and NRT Family

#### 2.4.1. Plasma-Membrane-Located Family Members, Function and the Effect of Their Expression Level Changes on the Cytosolic pH

Most AMTs (ammonium transporters) reported thus far are localized in the plasma membrane [86]. Amongst four distinct transport mechanisms in the AMTs family, NH_3_/H^+^ co-transport and NH_4_^+^/H^+^ symport are two mechanisms directly involving H^+^ transport [86]. Both TaAMT1;1 and AtAMT1;2 are NH_3_/H^+^ co-transporters [87,88], whereas PvAMT1;1 is a NH_4_^+^/H^+^ symporter [89]. All three proteins are proposed to be localized to the plasma membrane [89,90,91]. Consistent with its role in H^+^ influx across the plasma membrane, the expression of PvAMT1;1 in oocytes leads to a ~0.12 unit decrease in cytosolic pH [89]. Correspondingly, the expression of an NH_4_^+^ uniporter (LeAMT1;1) in oocytes has no effect on cytosolic pH [92].

Although possessing different substrates, the substrate transport mediated by most NRTs (nitrate transporters) shares a common feature, i.e., H^+^-coupling [93,94,95,96]. Electrophysiological results demonstrate that plasma membrane nitrate transporters such as BnNRT1.2 [97], AtNRT1.1 [98,99], AtNRT1.4 [100], AtNRT1.5 [101], AtNRT1.6 [102], OsNRT1 [103], and OsNRT2.3b [104] mediate H^+^/NO_3_^−^ symport, and the ratio of H^+^ is >1. Expression of OsNRT2.3b in oocytes leads to the ~0.16-unit acidification of cytoplasm [104]. In planta knockout of AtNRT1.1, the major molecular unit for nitrate uptake in Arabidopsis roots [105], causes a loss of alkalization of the growth medium and significantly reduces the adaptability of Arabidopsis to low-pH stress [106], supporting its contribution to cytosolic pH homeostasis by mediating H^+^ influx across the plasma membrane. AtNRT1.5 functions not only as a H^+^/NO_3_^−^ symporter, but also as a K^+^/H^+^ antiporter, mediating the efflux of K^+^ and an equivalent influx of H^+^ [107]. Both cases support its role in H^+^ influx, although the ratio of H^+^ is different (>1 for H^+^/NO_3_^−^ symporter; =1 for H^+^/K^+^ antiporter). Notably, substrate transport by some NRTs is not coupled to H^+^ [108], suggesting that not all plasma membrane NRTs confer H^+^ flux, a case similar to that of AMTs.

#### 2.4.2. Mechanism of H^+^ Transport

PvAMT1;1 functions as a NH_4_^+^/H^+^ symporter. H211E mutation results in the retaining of NH_4_^+^ transport, but the loss of H^+^ transport in this protein. All these results demonstrate that H211 is necessary for H^+^ transport in PvAMT1;1 [89]. Mutations of Q67H and W145S lead to the uncoupling of H^+^ transport from NH_3_/H^+^ transport in AtAMT1;2, indicating that the two residues (Q67 and W145) are essential for H^+^ transport in AtAMT1;2 [88].

The H^+^ transport in NRTs undergoes two steps. Firstly, proton receptor residues accept the proton in the outward-open conformation. Then, the transporters change into inward-open conformation and release H^+^ into the cytoplasm [109]. The crystal structure, in combination with mutation results, suggest that both the ExxER motif and a histidine residue confer H^+^ binding in plant NRTs [109,110,111,112,113]. AtNRT1.1 is the best structurally known plant NRT. Mutations of charged residues in its ExxER motif result in abolished H^+^ binding and NO_3_^−^ transport [110,111]. The crystal structure of AtNRT1.1 demonstrates that, in the outward-open conformation, one H^+^ is bound by the ExxER motif, and the other H^+^ is bound by the H356 [114].

#### 2.4.3. Regulation by Extracellular/Cytosolic pH

In agreement with their H^+^-coupled transport mechanism, extracellular acidification stimulates the transport activity of TaAMT1;1 [87] and PvAMT1;1 [89].

The observations from oocytes [115] and *Arabidopsis mutants* [105,106] indicate that the transport activity of AtNRT1.1 is enhanced by extracellular acidification. This extracellular-acid-stimulated transport seems a common feature of most plant plasma membrane NRTs, as detailed in Section 2.4.1 [97,98,99,100,101,102,103,104]. In contrast, a 0.16 pH unit (from 7.41 to 7.25) of cytosolic acidification arrests the nitrate transport activity of OsNRT2.3b. The amino acid residue H167 is necessary for this cytosolic pH regulation [104].

### 2.5. PHT Family

#### 2.5.1. Plasma-Membrane-Located Family Members, Function, and the Effect of Their Expression Level Changes on the Cytosolic pH

Amongst five clades of PHT (phosphate transporters) family, PHT1 is conceived to be the only subfamily that is localized to the plasma membrane. PHT1 contains many family members. It is reported that 9, 13, 13, and 11 PHT1 proteins are found in Arabidopsis, rice, maize, and barley genomes, respectively [116,117,118,119,120,121]. Direct subcellular localization experiments confirm that at least AtPHT1;1 [122], AtPHT1;2 [123], AtPHT1;4 [123], AtPHT1;9 [124], OsPHT1;3 [125], OsPHT1;4 [35,126], OsPHT1;8 [127], HvPHT1;1 [128], and HvPHT1;6 [129] are localized to the plasma membrane.

PHT1 subfamily mediates Pi uptake from the soil, and its transport mechanism is conceived to be H^+^-coupled H_2_PO_4_^−^ symport; the ratio between H^+^ and H_2_PO_4_^−^ is 2:1 to 4:1 [117,120,130,131]. Although the H^+^-coupled HPO_4_^2−^ (rather than H_2_PO_4_^−^) symport mechanism found in HvPHT1;6 challenges this consensus [129], the conclusion that substrate transport by PHT1 is coupled to H^+^ is unchanged. Consistent with its role in H^+^ influx across the plasma membrane, Pi uptake results in a ~0.2–0.3 unit decrease in cytosolic pH and corresponding alkalization of the growth medium in planta [132,133,134]. Expression of AtPHT1;9 in yeast leads to significant alkalization of the growth medium [124]. All these results indicate that PHT1 mediates H^+^ influx across the plasma membrane, and is finally involved in cytosolic pH homeostasis.

#### 2.5.2. Mechanism of H^+^ Transport

The crystal structure of PiPT from *Piriformospora indica* reveals that the proton is first received by D324, then transferred from the proton transport pathway that is constituted by D45, D48, E108, R139, and D149 residues, and finally released to the cytoplasm [135,136]. Homology modeling and point mutant results demonstrate that D35, D38, R134, and D144 (corresponding to D45, D48, R139, and D149) are essential for H^+^ transfer in AtPHT1;1 [137].

#### 2.5.3. Regulation by Extracellular/Cytosolic pH

When expressed in yeast, the transport activity of AtPHT1;1 is enhanced by extracellular acidification (pH gradually drops from 7.0 to 4.5) [137], whereas the activity of five rice PHT1 proteins exhibits a “bell-shaped” dependence on the extracellular pH. The optimum pH for the maximal transport activity is 6.5 in OsPHT1;1 [138] and OsPHT1;8 [127], 6.0 in OsPHT1;6 [139], and around 5.5–6.5 in OsPHT1;9 and OsPHT1;10 [140]. The difference in pH dependence amongst the above-mentioned PHT1 may be a result of the following. First, this distinct pH regulation strategy is an intrinsic property of PHT1. This is not surprising because even an H^+^-independent transport mechanism has been reported in another type of Pi transporter, PHO1 (PHOSPHATE 1) [141]. Second, the fact that the transport activity of five rice PHT1 proteins under different pH conditions is measured by the yeast growth rate (OD_600_), rather than direct Pi transport activity as shown in AtPHT1;1, may possibly cause an over-interpretation of the data. Thus, solid data from the direct Pi transport activity of PHT1 seem necessary for the clarification of their pH dependence.

### 2.6. KT/KUP/HAK Family

#### 2.6.1. Plasma-Membrane-Located Family Members, Function and the Effect of Their Expression Level Changes on the Cytosolic pH

Plant KT/HAK/KUPPHT (K^+^ transporter/high-affinity K^+^ transporter/K^+^ uptake permease) genes possess many family members. It is reported that 13, 27, and 27 KT/HAK/KUP genes are found in the genome of Arabidopsis, rice, and maize, respectively [142,143,144,145]. At the protein level, most KT/HAK/KUP proteins are conceived to be localized to the plasma membrane [143,146,147]. AtKUP1-12 and AtHAK5 are the names of 13 Arabidopsis KT/HAK/KUP [144]. Experimental evidence shows that AtHAK5 [148], AtKUP2 [149], AtKUP4 [150], AtKUP6 [151], and AtKUP7 [152] from Arabidopsis, and OsHAK1 [153,154], OsHAK5 [155,156], OsHAK19 [154], and OsHAK21 [157] from rice, are localized to the plasma membrane.

The fact that the high-affinity uptake of K^+^ in Arabidopsis root protoplasts [158] and in barley roots [159] is H^+^-coupled, and that AtHAK5 dominates the K^+^ uptake at less than 10 µM [148,160,161], indicate that AtHAK5 is most likely a K^+^/H^+^ symporter in planta [160,162,163]. This deduction is partially supported by the results from homologous proteins NcHAK1 of *Neurospora crassa* [164,165] and DmHAK5 of *Dionaea muscipula* [166], which are conceived as K^+^/H^+^ symporters, although further direct evidence is required (such as K^+^- and H^+^-dependent reversal potential shifts measured through electrophysiological experiments). Recently, crystal structure analysis of KimA (a plant KUP homologue) from *Bacillus subtilis* demonstrated that this protein functions as a K^+^/H^+^ symporter [167]. Thus, HAK5, and even the HAK family, is conceived to mediate H^+^ influx across the plasma membrane, finally contributing to the cytosolic pH homeostasis. Overexpression of OsHAK5 in rice results in the pH elevation of the growth medium [168].

#### 2.6.2. Mechanism of H^+^ Transport

The crystal structure, in combination with point mutation results, demonstrates that E233 confers H^+^ binding and release by its protonation and deprotonation in KimA (a plant KUP homologue from *Bacillus subtilis*) [167]. The conservation of this residue is expected to facilitate the understanding of H^+^ transport mechanisms in plant KT/KUP/HAK. Point mutation results show that the corresponding residue (E321) is essential for the transport activity of AtHAK5 [169].

#### 2.6.3. Regulation by Extracellular/Cytosolic pH

Extracellular acidification significantly stimulates the transport activity of plant KT/HAK/KUP [166,170,171], which is consistent with its putative role in K^+^/H^+^ symport.

## 3. Notable Issues in This Field

### 3.1. Not All Plasma Membrane Transporters Possess H^+^-Coupled Substrate Transport Mechanisms, and Using Transport Mechanisms of a Protein to Represent the Case of the Entire Family Is Not Suitable

The observation that nutrient uptake by plants is co-transported with H^+^ supports a long-standing hypothesis: transporters responsible for nutrient uptake are coupled with H^+^ [158,159,172,173,174]. However, as a result of in-depth study of the molecular elements of nutrient ion transport, increasing evidence shows that not all ion transporters are H^+^-coupled symporters and/or antiporters; examples are listed hereafter. First, four types of substrate transport mechanisms have been elucidated amongst AMTs [86]. Although H^+^/NH_4_^+^ symport (represented by PvAMT1;1) and H^+^/NH_3_ cotransport (represented by AtAMT1;2) are two types of mechanisms that are coupled to H^+^ [88,89], NH_3_ transport (represented by AtAMT2) and NH_4_^+^ uniport (represented by LeAMT1;1) serve as another two types of mechanisms that are H^+^-independent [175,176]. Second, regarding NRTs, although the majority of NRTs share a common feature, H^+^-coupled transport, an exception was found for AtNRT2.4, which mediates H^+^-uncoupled substrate transport [108]. Therefore, whether the transport is coupled with H^+^ is not a common feature of one transporter family, but a special characterization of one protein. Attempts to clarify the transport mechanisms of all family members only through the functional analysis of a protein are unsuitable. Additionally, intra-family members possess distinct structural bases for H^+^ transfer. For example, H356 is a key residue for H^+^ binding in AtNRT1.1, but this residue is not conserved between AtNRT1.5 and AtNRT1.8 [110]. As a conserved residue amongst AMTs, H211 is necessary for H^+^ transfer in PvAMT1;1. However, other intra-family members possessing this residue do not display similar H^+^-coupled transport, as shown in PvAMT1;1 [89]. The variation in structural basis for H^+^ transfer also indicates that H^+^ transport is an individual issue of transporter proteins.

### 3.2. Special Caution Is Needed When Drawing Conclusion to the H^+^ Transfer Mechanism of Transporters

The fact that transporter studies mainly focus on the transported ions, with less attention paid to the accompanied H^+^, objectively leads to the inappropriate interpretation of H^+^ transport. For example, first, several H^+^/substrate symport conclusions have been drawn just based on the observation that the transport activity of a protein is stimulated by extracellular acidification. Actually, functional enhancement by extracellular acidification may be the result of pH regulation. Second, H^+^ transport conclusions have been obtained just based on the linkage of a protein functional property with the results of early physiological measurements (root or protoplast) also seem unreasonable. That is because physiological measurement reflects the whole situation, whereas transporters responsible for this physiological response possibly possess a distinct transport mechanism regarding H^+^. Third, an H^+^ symport mechanism is proposed by the original literature based on insufficient experimental results; however, subsequent reference citations strengthen this hypothesis and give it the appearance of a truth. All these are disadvantageous to the study of the transmembrane transport of H^+^, which is an issue of physiological significance. Regarding the H^+^ transport of a transporter, we believe it should be supported by the following evidence: (1) hydrogen isotope labeling tests for yeast, *Xenopus* oocytes, and plant genetic materials (knockout and/or overexpression) harboring the target gene, or direct H^+^ flux measurements with technology such as non-invasive micro-tests, or extracellular/cytosolic pH measurements; (2) electrophysiological measurements. The pH regulation properties, as well as the reversal potential changes upon both the substrate and accompanying H^+^ concentration variations, should be contained, with the latter parameter facilitating the identification of H^+^ transport and calculation of the transport ratio between two ions; (3) third, perception of the crystal structure of transporters facilitates the understanding of the H^+^ transfer pathway; and (4) mutants with uncoupled H^+^ and substrate transport should be observed.

## 4. Roles of H^+^ Transport in Genetic Plant Improvements and Stress Resistance

### 4.1. Increasing Yield

H^+^ transport mediated by the above-mentioned proteins involves yield regulation. Examples are listed as follows.

Overexpression of OSA1 in rice significantly increases yield. One reason is that overexpression of this gene significantly enhances the ability of rice to excrete protons, which can not only ensure the homeostasis of cytosolic pH, but also form a stronger proton driving force and enhance the absorption of nutrients by the roots [35].

Overexpression of OsNRT2.3b in rice greatly promotes yield. One reason is that overexpression of this gene leads to phloem sap acidification, which facilitates the transport of P/Fe to the leaves [104].

Overexpression of OsHAK5 in rice notably increases yield. One reason is that overexpression of this gene leads to the alkalization of the extracellular medium, which facilitates the transport of IAA into the cytosol [168].

### 4.2. Acid Stress Resistance

H^+^ transport mediated by the above-mentioned proteins participates in acid stress resistance. Several lines of evidence are listed below.

Overexpression of an active form of H^+^-ATPase, AHA3-T498D in Arabidopsis, facilitates resistance to acid stress. This phenomenon is attributed to the enhanced excretion of H^+^ from the cytosol, favoring cytosolic pH homeostasis [34].

Overexpression of AtNRT1.1 in Arabidopsis significantly increases the resistance to acid stress. This observation is the result of the enhanced consumption of extracellular H^+^, creating a more favorable rhizosphere pH [177].

## 5. Conclusions and Prospects

**Figure 1:**
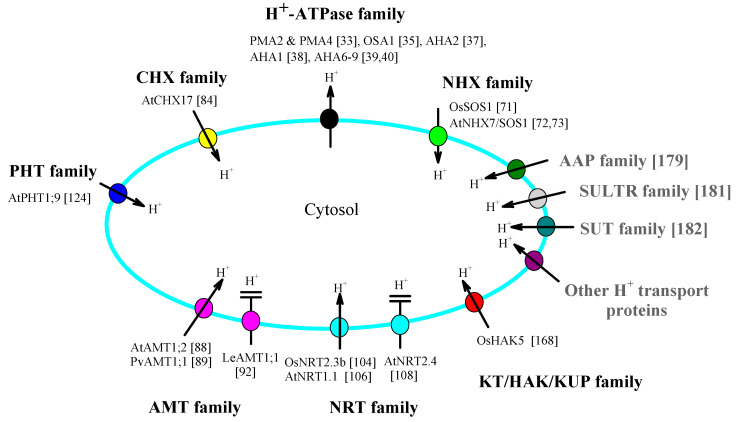
Plasma-membrane-located H^+^ transport proteins. H^+^-ATPase family functions in mediating H^+^ efflux from the cytosol to the extracellular space, whereas most members of the NHX, CHX, AMT, NRT, PHT, KT/HAK/KUP, AAP, SULTR, SUT family are responsible for mediating H^+^ influx from the extracellular space to the cytosol. Notably, several intra-family members of AMT and NRT do not transport H^+^, indicating that not all plasma membrane transporters possess H^+^-coupled substrate transport mechanisms. Seven types of H^+^ transport proteins focused on in this review (H^+^-ATPase, NHX, CHX, AMT, NRT, PHT, and KT/HAK/KUP) are indicated by black font, whereas other proteins (AAP, SULTR, SUT, etc.) are indicated by the gray font. Specific proteins with experimental evidences (references are indicated by [number]) are presented in the corresponding family. Arrows (**↑**) indicate the direction of H^+^ flux. Inability to transport H^+^ is indicated by special lines (symbols as shown for LeAMT1;1 and AtNRT2.4). Abbreviations: H^+^-ATPase (H^+^-pumping ATPase), NHX (Na^+^/H^+^ exchanger), CHX (cation/H^+^ exchanger), AMT (ammonium transporter), NRT (nitrate transporter), PHT (phosphate transporter), KT/HAK/KUP (K^+^ transporter/high-affinity K^+^ transporter/K^+^ uptake permease), SUT (Sucrose transporter), AAP (amino acid permease) and SULTR (sulfate transporter).

H^+^-ATPases and multiple transporters mediate H^+^ flux across the plasma membrane and are proposed to be essential for cytosolic pH homeostasis in plants. This review focused on seven types of proteins (H^+^-ATPase, NHX, CHX, AMT, NRT, and the KT/HAK/KUP family) that possess direct H^+^ transport activity, concentrating on the following four items: plasma-membrane-located family members, the effect of changes in their expression level on the cytosolic pH, the H^+^ transport pathway, and their functional regulation by the extracellular/cytosolic pH (summarized in Figure 1 and Table 1). Conclusions are drawn as follows. First, each of these seven types of protein is capable of mediating H^+^ flux across the plasma membrane, thus contributing to cytosolic pH homeostasis. However, intra-family members possess distinct H^+^ transport properties, with some members possessing the ability to transport H^+^, whereas other members are unable to transport H^+^. Second, the H^+^ transport activities of each of these seven types of protein are regulated by extracellular and cytosolic pH. However, intra-family members possess distinct pH regulation properties. Third, each of these seven types of protein has different H^+^ transport structural bases, and intra-family members possess different H^+^ transport structural bases.

We believe that the following points necessitate further attention. First, in view of the fact that intra-family members possess distinct H^+^ transport properties and underlying structural bases, using the transport mechanism of a protein to represent the case of the entire family is not suitable. Second, as an accompanying ion that is co-transported by most nutrient uptake transporters, H^+^ receives less attention, leading to the fact the conclusions drawn regarding their H^+^ transport are somewhat imprecise. Subsequent studies regarding H^+^ transport of related proteins should rely on much more solid evidence, which is proposed in Section 3. Third, the matter of how these proteins cooperate to achieve cytosolic pH homeostasis awaits further study [178]. Additionally, except for the seven types of protein, transporters such as H^+^-coupled sucrose transporters (abbreviated as SUT), H^+^-coupled amino acid permease (abbreviated as AAP), and sulfate transporters (abbreviated as SULTR) are also conceived to contribute to the cytosolic pH through direct mediating H^+^ flux across the plasma membrane [8,179,180,181,182]. Studies on these proteins, and the coordination of these plasma membrane H^+^ transport proteins, in addition to organelle-located ones, are crucial for the elucidation of the molecular mechanism for cytosolic pH homeostasis. Finally, in addition to maintaining cytoplasmic pH homeostasis, the physiological significance of H^+^ transport mediated by these proteins needs to be further explored, and several examples are provided in Section 4.

## Figures and Tables

**Table 1:** Functional regulation by extracellular and/or cytosolic pH and key residues for H^+^ transport.

Protein Name	Regulation by pH	Key Residues of H^+^ Transfer Pathway
**H^+^-ATPase family**		
AHA2	Bell-shaped dependence on cytosolic pH, with maximal transport activity approaching at pH 6.6 [47]	D684, N106 and R655 [29,41,42,43]
AHA1&AHA3, NpPMA2 &NpPMA4, and rice H^+^-ATPases	Bell-shaped dependence on cytosolic pH, with maximal transport activity approaching at pH 6.0–6.6 [33,40,45,46,47,48]	
AHA7	Active only when extracellular pH is ≥ 6.0 [44]	
**NHX family**		
AtNHX7/SOS1		Unclear [74,75]
PpSOS1	Stimulated by extracellular acidification [76]	
**CHX family**		
AtCHX13	Stimulated by extracellular acidification [79]	
AtCHX17		Unclear [84]
**AMT family**		
PvAMT1;1	Stimulated by extracellular acidification [89]	H211 [89]
AtAMT1;2		Q67, W145 [88]
**NRT family**		
AtNRT1.1	Stimulated by extracellular acidification [98,99,115]	(41)EXXER(45), H356 [110,111,114]
BnNRT1.2, AtNRT1.4, AtNRT1.5, AtNRT1.6, OsNRT1, OsNRT2.3b	Stimulated by extracellular acidification [97,100,101,102,103,104]	
OsNRT2.3b	Inhibited by cytosolic acidification [104]	
**PT family**		
AtPHT1;1	Stimulated by extracellular acidification [137]	D35, D38, R134 and D144 [137]
OsPHT1;1, OsPHT1;6, OsPHT1;8, OsPHT1;9, OsPHT1;10	Bell-shaped dependence on cytosolic pH, with maximal transport activity approaching at pH 5.5–6.5 [127,138,139,140]	
**KT/HAK/KUP family**		
AtHAK5		E312 [169]
DmHAK5, CnHAK1&CnHAK2, HvHAK1 & HvHAK2	Stimulated by extracellular acidification [166,170,171]	
